# Associations of Electronic Device Use Before and After Sleep With Psychological Distress Among Chinese Adults in Hong Kong: Cross-Sectional Study

**DOI:** 10.2196/15403

**Published:** 2020-06-11

**Authors:** Jung Jae Lee, Man Ping Wang, Tzu Tsun Luk, Ningyuan Guo, Sophia Siu-Chee Chan, Tai Hing Lam

**Affiliations:** 1 School of Nursing University of Hong Kong Hong Kong China (Hong Kong); 2 School of Public Health University of Hong Kong Hong Kong China (Hong Kong)

**Keywords:** addictive behavior, anxiety, computers, depression, devices, internet, smartphone, withdrawal symptoms

## Abstract

**Background:**

Hong Kong has a high rate of electronic device (e-device; computer, smartphone, and tablet) use. However, little is known about the associations of the duration of e-device use before and after sleep with psychological symptoms.

**Objective:**

This study aimed to investigate the associations of the duration of e-device use before and after sleep with psychological distress.

**Methods:**

A probability-based telephone survey was conducted on 3162 Hong Kong adults (54.6% female; mean age 47.4 years, SD 18.3 years) in 2016. Multivariate linear and Poisson regressions were used to calculate adjusted regression coefficients (aBs) and prevalence ratios (aPRs) of anxiety and depressive symptoms (measured by Patient Health Questionnaire-4) for the duration from waking to the first e-device use (≥61, 31-60, 6-30, and ≤5 minutes) and the duration of e-device use before sleeping (≤5, 6-30, 31-60, and ≥61 minutes).

**Results:**

The first e-device use in ≤5 (vs ≥61) minutes after waking was associated with anxiety (aB 0.35, 95% CI 0.24-0.46; aPR 1.74, 95% CI 1.34-2.25) and depressive symptoms (aB 0.27, 95% CI 0.18-0.37; aPR 1.84, 95% CI 1.33-2.54). Using e-devices for ≥61 (vs ≤5) minutes before sleeping was also associated with anxiety (aB 0.17, 95% CI 0.04-0.31; aPR 1.32, 95% CI 1.01-1.73) and depressive symptoms (aB 0.17, 95% CI 0.05-0.28; aPR 1.47, 95% CI 1.07-2.02). E-device use both ≤5 minutes after waking and for ≥61 minutes before sleeping was strongly associated with anxiety (aB 0.68, 95% CI 0.47-0.90; aPR 2.64, 95% CI 1.90-3.67) and depressive symptoms (aB 0.55, 95% CI 0.36-0.74; aPR 2.56, 95% CI 1.69-3.88).

**Conclusions:**

E-device use immediately (≤5 minutes) after waking and use for a long duration (≥61 minutes) before sleeping were associated with anxiety and depressive symptoms among Chinese adults in Hong Kong.

## Introduction

Electronic devices (e-devices), such as computers, smartphones, and tablets, are forms of information and communication technology (ICT) that have become essential to modern society [1,2]. The penetration rate of e-devices in Hong Kong, one of the most modern cities in China, is among the highest globally (eg, 80.2% for computers and 88.6% for smartphones) [3,4].

Given their popularity, the excessive use of e-devices, particularly smartphones, has been increasingly reported [1,5-8]. Despite ongoing debates about how to best define excessive use within the domain of addiction, the World Health Organization (WHO) [1] reported that excessive e-device use is arguably a type of behavioral addiction that presents as a repetitive pattern of behavioral engagement in a specific area (ie, e-device use), which eventually affects behavioral engagement in other domains [5,6,9]. Gaming disorders, which are closely associated with e-device use, have recently been included in the WHO’s 11th Revision of the International Classification of Diseases (ICD-11) [9].

Increasing concerns have emerged about the adverse psychological effects of behavioral addiction with e-devices [8,10-17]. A recent systematic review found that e-device addiction was associated with anxiety (β coefficients, .12-.23) and depression (β coefficients, .15-.48) [15]. These findings were supported by another recent study finding that behavioral addiction to the internet and smartphones resulted in the imbalance of gamma-aminobutyric acid levels in the brain, which has been implicated in the development of depression and anxiety [18].

The WHO has urged the consideration of e-device use duration when defining behavioral addiction with e-devices [1]. Many people start and end their days with e-devices owing to their ubiquity and convenience [19]. Borrowing the concept of adopting the duration of the first tobacco use after waking up in the morning to assess tobacco addiction [20], the duration of the first e-device use may indicate the level of e-device addiction. As negative sleep outcomes, such as shortened sleep duration and sleep onset latency, are associated with excessive bedside e-device use [8,21-23], it is hypothesized that a longer duration of e-device use before sleep (ie, longer screen time before sleep) may also be associated with excessive use and behavioral addiction.

Little is known about the associations of the duration of e-device use before and after sleep with psychological symptoms. Most existing studies have focused on identifying the associations between psychological symptoms and the addiction of e-devices or on developing assessment instruments for e-device addiction [8,10-17]. In addition, most studies have focused only on young age groups, despite the rapid adoption of e-devices among older age groups [24]. Therefore, we aimed to investigate the association of psychological distress with the duration of e-device use before and after sleep in Hong Kong adults.

## Methods

### Study Design

The Hong Kong Family and Health Information Trends Survey (FHInTS) is part of the FAMILY project (A Jockey Club Initiative for a Harmonious Society project). The project has conducted periodic surveys to study family and individual health in relation to the use of ICTs in Hong Kong since 2009. The current FHInTS was the fifth edition of the project, and it explored the areas of ICT use for family health, health communication, and information acquisition [25]. Probability-based telephone surveys of individuals from the general public aged 18 years or older, who had at least one e-device and spoke Cantonese (ie, sample selection criteria), were conducted from January to August 2016 by trained interviewers of the Public Opinion Programme at the University of Hong Kong, a local reputable survey agency.

A random sampling method with two phases was adopted for the survey. Hong Kong’s residential telephone directories were used to extract telephone numbers. The last digit of the extracted numbers was converted by adding or subtracting 1 or 2 to make up unlisted numbers in the directories. The telephone numbers were randomly listed using computer software (ie, first phase random sampling). In the second phase, the nearest birthday rule was used to randomly select a respondent [26]. To minimize selection bias by including individuals who work away from home during the day, the telephone survey was mostly conducted in the evenings. Verbal informed consent was collected from each participant over the telephone, and each telephone survey lasted for about 20 minutes. Ethical approval was obtained from the Institutional Review Board of The University of Hong Kong/Hospital Authority Hong Kong West Cluster.

### Measurements

Respondents were asked two questions to obtain the following data that were considered as duration indicators of e-device use: (1) duration from waking to the first e-device use (how long after getting up in the morning do you usually start using your computer, tablet, or smartphone? [response options were ≥61, 31-60, 6-30, and ≤5 minutes]) and (2) duration of e-device use before sleeping (how much time do you spend on using your computer, tablet, or smartphone before sleeping? [response options were ≤5, 6-30, 31-60, and ≥61 minutes]). Duration-related questions have been asked in other e-device addiction studies [13].

Anxiety and depressive symptoms were measured using Patient Health Questionnaire-4 (PHQ-4) [27], a brief self-reported instrument combining Generalized Anxiety Disorder-2 (GAD-2) [28] and Patient Health Questionnaire-2 (PHQ-2) [29] for screening anxiety and depression. GAD-2 and PHQ-2 scores of 3 or above on a scale of 0 to 6 were considered to indicate high risks of anxiety and depression [27]. The Cronbach α of PHQ-4 was .82, and the values of the PHQ-2 and GAD-2 subscales in this study were .71 and .75, respectively, which were considered satisfactory given their brevity. The score of PHQ-4 correlated well with the score of Perceived Stress Scale-4 [30] in a random subsample of 792 participants (*r*=0.61, *P*<.001), supporting its convergent validity.

### Statistical Analysis

All data were weighted by age, sex, and education level distributions according to the Hong Kong census data from 2015 [31]. The mean scores of anxiety and depressive symptoms related to sociodemographic characteristics, including sex, age, education level, and household income, and the duration from waking to the first e-device use as well as the duration of e-device use before sleeping were compared using the independent sample *t* test or one-way analysis of variance (ANOVA), as appropriate. The Spearman rank correlation coefficient was calculated to analyze the correlation between the duration from waking to the first e-device use and the duration of e-device use before sleeping. A linear regression was used to compute adjusted regression coefficients (aBs) of anxiety and depressive symptoms for the duration from waking to the first e-device use and the duration of e-device use before sleeping. Age, sex, highest education level, and monthly household income were included in the adjusted regression models. Similar to the linear regression, Poisson regression with a robust variance estimator [32] was used to calculate the adjusted prevalence ratios (aPRs) of risk for anxiety and depression in terms of different durations of e-device use after waking and before sleeping, adjusting for age, sex, education level, and household income. As subgroup analyses, we included multiplicative interaction terms of the participants’ durations of e-device use with each demographic in the regression models to compute *P* values for interaction effects. A sensitivity test was performed by repeating multivariate linear regression models using the PHQ-4 score as the outcome variable. Available case analysis was used to handle missing data, as the proportion of missing data was very low. Stata version 15 (StataCorp) was used for all analyses, and a *P* value <.05 was regarded as statistically significant.

## Results

### Participant Characteristics

Among 6890 adults eligible for the FHInTS, who were contacted, 5080 successfully completed the interviews (response rate 73.73%). Of these 5080 respondents, 3162 (62.24%) possessed at least one e-device and were included in the present study. The mean age of the respondents was 47.40 years (SD 18.32). Additionally, 54.55% (weighted 1833/3361) were female, 79.77% (2681/3361) were aged 25 to 64 years, 85.66% (2879/3361) had secondary or higher education, and 68.42% (2058/3008) had a monthly household income of HK $20,000 (US $2564; US $1=HK $7.80) or more (Table 1).

**Table 1:** Weighted mean scores of anxiety and depressive symptoms by sociodemographic characteristics in Hong Kong adults who used e-devices.

Characteristic		Value (N=3162), n (%^a^)		Weighted value (N=3361), n (%^a^)		Anxiety symptoms (GAD-2^b^ score; (mean 1.10, SD 0.02)					Depressive symptoms (PHQ-2^c^ score; mean 0.72, SD 0.02)		
						Score		*P* value^d^		Score		*P* value^d^	
**Sex**								.01				.76	
	Male		1226 (38.77)		1528 (45.45)		1.04				0.75		
	Female		1936 (61.23)		1833 (54.55)		1.13				0.71		
**Age** **(years)**								<.001				<.001	
	18-24		527 (16.67)		382 (11.36)		1.67				1.25		
	25-34		325 (10.28)		695 (20.68)		1.50				1.04		
	35-44		393 (12.43)		701 (20.87)		1.23				0.80		
	45-54		599 (18.94)		725 (21.56)		1.03				0.61		
	55-64		715 (22.61)		560 (16.66)		0.81				0.49		
	≥65		603 (19.07)		298 (8.88)		0.69				0.42		
**Education**								<.001				.11	
	Primary or less		387 (12.24)		482 (14.34)		0.79				0.54		
	Secondary		1452 (45.92)		1766 (52.55)		1.00				0.70		
	Tertiary or greater		1323 (41.84)		1113 (33.11)		1.29				0.79		
**Monthly household income^e^ (HK $)**								.13				.01	
	≤19,999		961 (34.07)		950 (31.58)		1.11				0.77		
	20,000-39,999		928 (32.90)		1065 (35.40)		1.08				0.74		
	≥40,000		932 (33.04)		993 (33.02)		1.09				0.64		
**Duration from waking to the first e-device use (four categories)**								<.001				<.001	
	≥61 min		1418 (45.02)		1409 (42.21)		0.92				0.58		
	31-60 min		389 (12.35)		452 (13.53)		1.06				0.77		
	6-30 min		628 (19.94)		684 (20.49)		1.09				0.74		
	≤5 min		715 (22.70)		793 (23.77)		1.47				0.98		
**Duration from waking to the first e-device use (two categories)**								<.001				<.001	
	≥6 min		2435 (77.30)		2544 (76.23)		0.99				0.65		
	≤5 min		715 (22.70)		793 (23.77)		1.47				0.98		
**Duration of e-device use before sleeping (four categories)**								<.001				<.001	
	≤5 min		1117 (35.51)		1009 (30.26)		0.82				0.50		
	6-30 min		1033 (32.84)		1162 (34.85)		1.16				0.74		
	31-60 min		559 (17.77)		661 (19.81)		1.27				0.93		
	≥61 min		437 (13.89)		503 (15.08)		1.43				0.98		
**Duration of e-device use before sleeping (two categories)**								<.001				<.001	
	≤60 min		2709 (86.11)		2832 (84.92)		1.04				0.68		
	≥61 min		437 (13.89)		503 (15.08)		1.43				0.98		
**Combined duration^f^**								<.001				<.001	
	≥6W^g^ & ≤60S^h^		2144 (68.37)		2206 (66.46)		0.95				0.62		
	≥6W^g^ & ≥61S^h^		281 (8.96)		325 (9.78)		1.24				0.86		
	≤5W^g^ & ≤60S^h^		555 (17.70)		610 (18.39)		1.40				0.92		
	≤5W^g^ & ≥61S^h^		156 (4.97)		178 (5.37)		1.76				1.19		

^a^Calculated percentages were rounded off to two decimal places, accordingly combined percentages can exceed 100%.

^b^GAD-2: Generalized Anxiety Disorder Questionnaire-2.

^c^PHQ-2: Patient Health Questionnaire-2.

^d^*P* for *t* test (two groups) or ANOVA (three groups or above).

^e^US $1=HK $7.8.

^f^Duration from waking to the first e-device use plus duration of e-device use before sleeping (two categorizations).

^g^6W and 5W denote the duration from waking to the first e-device use for 6 minutes and 5 minutes, respectively.

^h^60S and 61S denote the duration of e-device use before sleeping for 60 minutes and 61 minutes, respectively.

### Associations of Participant Characteristics With Psychological Distress

Anxiety symptoms were associated with being female (*P*=.01), being younger (*P*<.001), and having higher education levels (*P*<.001), whereas depressive symptoms were associated with being younger (*P*<.001) and having lower income (*P*=.01) (Table 1). Respondents who first used e-devices for shorter durations of time after waking had more anxiety and depressive symptoms (*P*<.001). Similarly, respondents who used e-devices for longer periods of time before sleeping had more anxiety and depressive symptoms (*P*<.001). Respondents who first used e-devices within shorter periods of time after waking and for longer periods of time before sleeping had more anxiety and depressive symptoms as compared with other e-device use patterns (*P*<.001).

### Associations of E-Device Use Before and After Sleep With Psychological Distress

The duration of e-device use before sleeping was significantly correlated with the duration from waking to the first e-device use (*r*_s_=0.304, *P*<.001). Shorter periods of time between waking and e-device use (ie, ≤5 minutes) were associated with both anxiety (aB 0.35, 95% CI 0.24-0.46) and depressive symptoms (aB 0.27, 95% CI 0.18-0.37) (Tables 2 and 3). Shorter durations of time before e-device use were consistently associated with both anxiety (aPR 1.74, 95% CI 1.34-2.25) and depressive symptoms (aPR 1.84, 95% CI 1.33-2.54) (Tables 4 and 5). Longer durations of e-device use before sleeping (ie, ≥61 minutes) were associated with higher risks of both anxiety (aB 0.17, 95% CI 0.04-0.31; aPR 1.32, 95% CI 1.01-1.73) and depressive symptoms (aB 0.17, 95% CI 0.05-0.28; aPR 1.47, 95% CI 1.07-2.02). The use of e-devices starting ≤5 minutes after waking and for ≥61 minutes before sleeping showed the strongest association with anxiety and depressive symptoms (aB 0.68 and aPR 2.64 for anxiety symptoms; aB 0.55 and aPR 2.56 for depressive symptoms). The results from the sensitivity test, which examined the association of e-device use before and after sleep with PHQ-4 scores, yielded similar results, thus supporting the robustness of the findings.

**Table 2:** Linear regression of anxiety symptoms for the duration from waking to the first e-device use and duration of e-device use before sleeping.

Duration assessed and time (min)^a^		Anxiety symptoms (GAD-2^b^ score)			
		Crude regression coefficient (95% CI)	*P*	Adjusted^c^ regression coefficient (95% CI)	*P*
**Duration from waking to the first e-device use**					
	≥61	0		0	
	31-60	0.03 (−0.11 to 0.17)	.69	−0.10 (−0.25 to 0.04)	.17
	6-30	0.07 (−0.05 to 0.20)	.24	−0.09 (−0.22 to 0.04)	.18
	≤5	0.45 (0.34 to 0.57)	<.001	0.31 (0.18 to 0.43)	<.001
	Trend		<.001		<.001
	≥6	0		0	
	≤5	0.43 (0.32 to 0.54)	<.001	0.35 (0.24 to 0.46)	<.001
**Duration of e-device use before sleeping**					
	≤5	0		0	
	6-30	0.26 (0.15 to 0.38)	<.001	0.04 (−0.09 to 0.16)	.54
	31-60	0.41 (0.27 to 0.54)	<.001	0.16 (0.01 to 0.31)	.03
	≥61	0.54 (0.40 to 0.68)	<.001	0.23 (0.07 to 0.39)	.004
	Trend		<.001		.002
	≤60	0		0	
	≥61	0.34 (0.21 to 0.47)	<.001	0.17 (0.04 to 0.31)	.01
**Duration from waking to the first e-device use plus duration of e-device use before sleeping (two categorizations)**					
	≥6W^d^ & ≤60S^e^	0		0	
	≥6W^d^ & ≥61S^e^	0.19 (0.04 to 0.35)	.02	−0.01 (−0.18 to 0.15)	.87
	≤5W^d^ & ≤60S^e^	0.35 (0.23 to 0.47)	<.001	0.26 (0.13 to 0.38)	<.001
	≤5W^d^ & ≥61S^e^	0.82 (0.61 to 1.03)	<.001	0.68 (0.47 to 0.90)	<.001
	Trend		<.001		<.001

^a^All data were weighted by sex, age, and education level distribution of the Hong Kong general population.

^b^GAD-2: Generalized Anxiety Disorder Questionnaire-2.

^c^Adjusted for age, sex, highest education level, and monthly household income.

^d^6W and 5W denote the duration from waking to the first e-device use for 6 minutes and 5 minutes, respectively.

^e^60S and 61S denote the duration of e-device use before sleeping for 60 minutes and 61 minutes, respectively.

**Table 3:** Linear regression of depressive symptoms for the duration from waking to the first e-device use and duration of e-device use before sleeping.

Duration assessed and time (min)^a^		Depressive symptoms (PHQ-2^b^ score)			
		Crude regression coefficient (95% CI)	*P*	Adjusted^c^ regression coefficient (95% CI)	*P*
**Duration from waking to the first e-device use**					
	≥61	0		0	
	31-60	0.11 (−0.02 to 0.24)	.09	0.04 (−0.09 to 0.17)	.52
	6-30	0.09 (−0.02 to 0.20)	.09	−0.01 (−0.12 to 0.11)	.92
	≤5	0.40 (0.30 to 0.50)	<.001	0.28 (0.17 to 0.39)	<.001
	Trend		<.001		<.001
	≥6	0		0	
	≤5	0.36 (0.26 to 0.45)	<.001	0.27 (0.18 to 0.37)	<.001
**Duration of e-device use before sleeping**					
	≤5	0		0	
	6-30	0.16 (0.06 to 0.26)	.001	0.01 (−0.10 to 0.13)	.79
	31-60	0.41 (0.29 to 0.52)	<.001	0.19 (0.06 to 0.32)	.005
	≥61	0.44 (0.31 to 0.57)	<.001	0.22 (0.08 to 0.37)	.002
	Trend		<.001		<.001
	≤60	0		0	
	≥61	0.28 (0.16 to 0.39)	<.001	0.17 (0.05 to 0.28)	.006
**Duration from waking to the first e-device use plus duration of e-device use before sleeping (two categorizations)**					
	≥6W^d^ & ≤60S^e^	0		0	
	≥6W^d^ & ≥61S^e^	0.16 (0.02 to 0.30)	.02	0.03 (−0.11 to 0.18)	.67
	≤5W^d^ & ≤60S^e^	0.30 (0.19 to 0.41)	<.001	0.20 (0.09 to 0.31)	<.001
	≤5W^d^ & ≥61S^e^	0.67 (0.49 to 0.85)	<.001	0.55 (0.36 to 0.74)	<.001
	Trend		<.001		<.001

^a^All data were weighted by sex, age, and education level distribution of the Hong Kong general population.

^b^PHQ-2: Patient Health Questionnaire-2.

^c^Adjusted for age, sex, highest education level, and monthly household income.

^d^6W and 5W denote the duration from waking to the first e-device use for 6 minutes and 5 minutes, respectively.

^e^60S and 61S denote the duration of e-device use before sleeping for 60 minutes and 61 minutes, respectively.

**Table 4:** Poisson regression of anxiety symptoms for the duration from waking to the first e-device use and duration of e-device use before sleeping.

Duration assessed and time (min)^a^		Value, n (%)	Anxiety symptoms (GAD-2^b^ score ≥3)^c^			
			Crude prevalence ratio (95% CI)	*P*	Adjusted^d^ prevalence ratio (95% CI)	*P*
**Duration from waking to the first e-device use**						
	≥61	139 (9.88)	1		1	
	31-60	44 (9.84)	1.00 (0.66-1.50)	.98	0.81 (0.52-1.27)	.36
	6-30	60 (8.75)	0.89 (0.62-1.26)	.50	0.71 (0.48-1.04)	.08
	≤5	135 (16.99)	1.72 (1.30-2.27)	<.001	1.51 (1.12-2.04)	.007
	Trend			.002		.04
	≥6	218 (9.59)	1		1	
	≤5	135 (16.99)	1.78 (1.39-2.27)	<.001	1.74 (1.34-2.25)	<.001
**Duration of e-device use before sleeping**						
	≤5	90 (8.89)	1		1	
	6-30	130 (11.23)	1.26 (0.92-1.73)	.14	1.00 (0.70-1.42)	.99
	31-60	76 (11.49)	1.29 (0.91-1.83)	.15	0.98 (0.66-1.47)	.92
	≥61	82 (16.22)	1.82 (1.28-2.60)	.001	1.31 (0.88-1.97)	.18
	Trend			.002		.25
	≤60	296 (10.46)	1		1	
	≥61	82 (16.22)	1.55 (1.16-2.08)	.003	1.32 (0.96-1.82)	.09
**Duration from waking to the first e-device use plus duration of e-device use before sleeping (two categorizations)**						
	≥6W^e^ & ≤60S^f^	208 (9.45)	1		1	
	≥6W^e^ & ≥61S^f^	31 (9.69)	1.03 (0.65-1.62)	.91	0.74 (0.48-1.16)	.26
	≤5W^e^ & ≤60S^f^	85 (13.87)	1.47 (1.10-1.95)	.009	1.36 (1.03-1.79)	.047
	≤5W^e^ & ≥61S^f^	50 (28.11)	2.98 (2.10-4.22)	<.001	2.64 (1.90-3.67)	<.001
	Trend			<.001		<.001

^a^All data were weighted by sex, age, and education level distribution of the Hong Kong general population.

^b^GAD-2: Generalized Anxiety Disorder Questionnaire-2.

^c^High prevalence of anxiety.

^d^Adjusted for age, sex, highest education level, and monthly household income.

^e^6W and 5W denote the duration from waking to the first e-device use for 6 minutes and 5 minutes, respectively.

^f^60S and 61S denote the duration of e-device use before sleeping for 60 minutes and 61 minutes, respectively.

**Table 5:** Poisson regression of depressive symptoms for the duration from waking to the first e-device use and duration of e-device use before sleeping.

Duration assessed and time (min)^a^			Value, n (%)		Depressive symptom (PHQ-2^b^ score ≥3)^c^						
					Crude prevalence ratio (95% CI)		*P*		Adjusted^d^ prevalence ratio (95% CI)		*P*
**Duration from waking to the first e-device use**											
	≥61	85 (6.04)		1				1			
	31-60	24 (5.36)		0.89 (0.53-1.49)		.65		0.80 (0.45-1.44)		.46	
	6-30	46 (6.72)		1.11 (0.71-1.74)		.64		1.10 (0.68-1.79)		.69	
	≤5	98 (12.33)		2.04 (1.43-2.92)		<.001		1.83 (1.23-2.72)		.003	
	Trend					<.001				.005	
	≥6	137 (6.04)		1				1			
	≤5	98 (12.33)		2.02 (1.49-2.75)		<.001		1.84 (1.33-2.54)		<.001	
**Duration of e-device use before sleeping**											
	≤5	56 (5.53)		1				1			
	6-30	69 (5.93)		1.07 (0.70-1.65)		.75		0.77 (0.47-1.26)		.29	
	31-60	71 (10.69)		1.93 (1.25-2.99)		.003		1.21 (0.73-2.03)		.46	
	≥61	54 (10.78)		1.95 (1.23-3.10)		.005		1.40 (0.83-2.36)		.21	
	Trend					<.001				.049	
	≤60	195 (6.90)		1				1			
	≥61	54 (10.78)		1.56 (1.08-2.26)		.02		1.47 (1.07-2.02)		.02	
**Duration from waking to the first e-device use plus duration of e-device use before sleeping (two categorizations)**											
	≥6W^e^ & ≤60S^f^	127 (5.78)		1				1			
	≥6W^e^ & ≥61S^f^	24 (7.49)		1.30 (0.77-2.17)		.32		1.18 (0.74-1.87)		.48	
	≤5W^e^ & ≤60S^f^	68 (11.13)		1.93 (1.35-2.75)		<.001		1.66 (1.19-2.31)		.003	
	≤5W^e^ & ≥61S^f^	30 (16.78)		2.91 (1.79-4.73)		<.001		2.56 (1.69-3.88)		<.001	
	Trend					<.001				<.001	

^a^All data were weighted by sex, age, and education level distribution of the Hong Kong general population.

^b^PHQ-2: Patient Health Questionnaire-2.

^c^High prevalence of depression.

^d^Adjusted for sex, age, highest education level, and monthly household income.

^e^6W and 5W denote the duration from waking to the first e-device use for 6 minutes and 5 minutes, respectively.

^f^60S and 61S denote the duration of e-device use before sleeping for 60 minutes and 61 minutes, respectively.

### Interaction Effect Between Demographics and E-Device Use for Psychological Distress

Subgroup analyses showed that the associations of e-device use before and after sleep with psychological distress differed according to the participants’ demographics. Participants who were female, were aged 18 to 44 years, received secondary education or below, or had household income of HK $39,999 or less showed stronger associations in all but two outcomes; participants who received tertiary education and used e-devices starting ≤5 minutes after waking showed stronger associations with anxiety symptoms, whereas the association between female participants who used e-devices for ≥61 minutes before sleeping and depressive symptoms was not relevant (Tables 6 and 7).

**Table 6:** Interaction effect between Hong Kong adults’ demographics and e-device use for anxiety symptoms.

Characteristic^a^		Duration from waking to the first e-device use (≥6 min vs ≤5 min)				Duration of e-device use before sleeping (≤60 min vs ≥61 min)			
		n	Coefficient^b^	*P* ^c^	n		Coefficient^b^	*P* ^c^	
**Sex**				<.001				.002	
	Men	1370	0.18 (0.02 to 0.34)		1365		0.10 (−0.07 to 0.28)		
	Women	1629	0.47 (0.31 to 0.62)		1628		0.23 (0.03 to 0.44)		
**Age (years)^d,e^**				<.001				<.001	
	18-44	1574	0.47 (0.32 to 0.62)		1572		0.26 (0.09 to 0.44)		
	≥45	1425	0.23 (0.06 to 0.39)		1422		0.07 (−0.14 to 0.28)		
**Education^d,f^**				<.001				<.001	
	Secondary or less	1984	0.28 (0.14 to 0.43)		1983		0.25 (0.07 to 0.42)		
	Tertiary or more	1015	0.45 (0.27 to 0.62)		1011		0.08 (−0.12 to 0.29)		
**Income (HK$)^d,g,h^**				<.001				<.001	
	≤39,999	2006	0.45 (0.30 to 0.59)		2009		0.24 (0.07 to 0.42)		
	≥40,000	993	0.21 (0.04 to 0.38)		985		0.06 (−0.14 to 0.27)		

^a^All data were weighted by sex, age, and education level distribution of the Hong Kong general population.

^b^Regression coefficient (95% CI) adjusted for age, sex, highest education level, and monthly household income.

^c^*P* value for interaction.

^d^Demographic variables were summarized into binary groups.

^e^The median age of the participants was 47 years.

^f^66.9% and 33.1% of the Hong Kong population attained secondary education or below and tertiary education, respectively, in 2018.

^g^The median monthly household income of economically active households in Hong Kong was around HK $36,000 in 2019.

^h^Monthly household income (US $1=HK $7.8).

**Table 7:** Interaction effect between Hong Kong adults’ demographics and e-device use for depressive symptoms.

Characteristic^a^		Duration from waking to the first e-device use (≥6 min vs ≤5 min)			Duration of e-device use before sleeping (≤60 min vs ≥61 min)		
		n	Coefficient^b^	*P* ^c^	n	Coefficient^b^	*P* ^c^
**Sex**				.004			.07
	Men	1371	0.21 (0.07 to 0.36)		1366	0.06 (−0.01 to 0.22)	
	Women	1629	0.31 (0.17 to 0.44)		1629	0.04 (−0.00 to 0.07)	
**Age (years)^d,e^**				<.001			<.001
	18-44	1574	0.36 (0.22 to 0.51)		1572	0.27 (0.11 to 0.44)	
	≥45	1426	0.19 (0.06 to 0.32)		1423	0.03 (−0.14 to 0.20)	
**Education^d,f^**				<.001			.03
	Secondary or less	1984	0.28 (0.15 to 0.41)		1984	0.20 (0.05 to 0.36)	
	Tertiary or more	1015	0.27 (0.12 to 0.43)		1011	0.11 (−0.07 to 0.29)	
**Income (HK $)^d,g,h^**				<.001			<.001
	≤39,999	2007	0.42 (0.29 to 0.55)		2010	0.26 (0.11 to 0.42)	
	≥40,000	993	0.03 (−0.12 to 0.17)		985	0.00 (−0.18 to 0.18)	

^a^All data were weighted by sex, age, and education level distribution of the Hong Kong general population.

^b^Regression coefficient (95% CI) adjusted for age, sex, highest education level, and monthly household income.

^c^*P* value for interaction.

^d^Demographic variables were summarized into binary groups.

^e^The median age of the participants was 47 years.

^f^66.9% and 33.1% of the Hong Kong population attained secondary education or below and tertiary education, respectively, in 2018.

^g^The median monthly household income of economically active households in Hong Kong was around HK $36,000 in 2019.

^h^Monthly household income (US $1=HK $7.8).

## Discussion

### Principal Findings

With a large population-representative sample of adult e-device users in Hong Kong, we found that both shorter time to the first e-device use and longer duration of e-device use before sleeping were associated with anxiety and depressive symptoms. The associations tended to be stronger in females, those aged 18 to 44 years, those who received secondary education or below, and those who had household income of HK $39,999 or less. To our knowledge, no study has yet presented the associations reported herein. Additionally, this research provides findings of Chinese adults’ e-device use in response to the dearth of e-device research in adult populations, particularly those of Chinese ethnicity.

### Comparison With Prior Work

While many studies [8,33-37] have consistently reported that e-device addiction or excessive e-device use is associated with anxiety and depressive symptoms, our study demonstrated that the use of an e-device for more than 60 minutes before sleeping increased the risk of depression and anxiety. Studies have established that excessive e-device use before sleeping (ie, ≥61 min) can affect sleep quality. It has been found that the emission of electromagnetic radiation from e-devices impairs the circadian rhythm, and the blue light emitted by e-devices affects melatonin secretion [38-40]. Particularly, one study found that the use of e-devices for over 60 minutes in the evening greatly hindered melatonin secretion at night (mean 48%, SD 4%) [40], resulting in poor sleep quality and quantity. Similarly, Hysing et al [22] asserted that using e-devices in the last hour (ie, 60 minutes) before sleeping prolonged sleep onset latency and increased sleep deficiency. Sleep quality [41-43] and melatonin secretion [44-46] are both negatively associated with anxiety and depressive symptoms. Therefore, it can be deduced that poor sleep quantity and quality owing to reduced melatonin secretion from over 60 minutes of e-device use before sleeping, in addition to prolonged exposure to its electromagnetic radiation, can potentially lead to psychological distress. The overall evidence so far suggests that e-devices should not be used for more than 60 minutes before bedtime, and whether such a practice can enhance sleep quality should be investigated.

A high number of public surveys [19,47-49] reported on the pattern of the first e-device use soon after waking as a symptom of the excessive use of e-devices, using descriptive data for the duration. For example, a global survey reported that 33% of people in developed countries, ranging from 20% in France to 64% in South Korea, used smartphones within 5 minutes of waking [47]. In this study, 22.7% (715/3162) of participants used an e-device within 5 minutes of waking, which is similar to the rate reported in France. Haug et al [13] identified that the duration from waking to the first e-device use was a strong indicator of e-device addiction. In addition to the association between e-device use in the morning and e-device addiction, our study showed that the duration from waking to the first e-device use was inversely associated with psychological symptoms.

We identified that there was a strong correlation between the duration from waking to the first e-device use and the duration of e-device use before sleeping. We also identified that the combination of e-device use ≤5 minutes after waking and ≥61 minutes before sleeping showed the highest risk and very high risk for anxiety and depressive symptoms, respectively, as compared with other combinations of e-device use duration. E-device use (particularly with the internet) at bedtime could affect e-device use in the morning. The behavior to immediately use e-devices upon waking is seen as a withdrawal symptom caused by excessive e-device use at bedtime [37,50,51]. In other words, late e-device use at bedtime would cause the first thought on one’s mind to use the device again upon waking up as a withdrawal symptom. Lin et al [50] also stressed that a prominent withdrawal behavior of e-device addiction is the pattern of e-device use upon waking (ie, an “eye opener”). The pattern would be a release of the craving to be continuously stimulated by real-time interactions via the e-device (or the internet) immediately upon opening one’s eyes in the morning. The “eye opener” concept has also been used to describe withdrawal symptoms of substance use (eg, alcohol and tobacco) in the literature [20,52].

We found that the association between the duration of e-device use and psychological distress differed according to participants’ demographics. Although it would be difficult to directly compare our study with existing studies as our findings are novel, some studies have reported that problematic e-device use has been more commonly found among female individuals [13,53-55], younger individuals [13,56,57], those with low education levels [56,57], and those with low household income [56,58,59], which is consistent with our findings. However, the associations were inconsistent in some other studies. For example, de-Sola et al [57] reported that being male had a higher correlation with problematic e-device use than being female, whereas Kim et al [60] found no sex and household income differences. Long et al [12] found that household income was positively associated with problematic e-device use. Hence, a meta-analysis will be useful to affirm the associations of the duration of e-device use and psychological distress with different demographic groups in populations. A qualitative study will also be required to obtain an in-depth understanding of these associations within the Hong Kong context.

Although excessive e-device use is associated with negative health effects, including psychological symptoms, avoiding the use of e-devices cannot be recommended as they are integrated into the daily life of humans in this era owing to their benefits (eg, enhanced communication [61]). Nurturing healthy patterns of device use should be advised. Given that a large number of respondents used e-devices upon waking and before sleeping, public education should be provided for the association between excessive “bedside” e-device use and negative psychological symptoms. Seo et al [18] also identified that a psychosocial intervention could rectify the chemical imbalances in the brain and reduce the negative psychological distress caused by the excessive use of e-devices. Health care professionals can also consider using brief duration indicators to screen for psychological distress among individuals in clinical and community settings.

### Limitations

Our study had several limitations. First, this study only included the e-devices of computers, smartphones, and tablets, and thus, it omitted the use of other e-devices, such as televisions and video game consoles, although most of the content would have involved computers or smartphones. Second, we conducted a probability-based telephone survey. The lack of mobile phone survey data may cause sampling bias. Third, the type of e-device used and the type of online activities conducted on e-devices may affect the durations of e-device use before and after sleep and associated psychological distress. Additional studies would be useful to assess these associations. Fourth, the causal relationships between respondents’ use of e-devices and psychological symptoms (eg, psychological distress induces excessive use and vice versa [15]) were uncertain owing to the cross-sectional study design [62]. Fifth, the respondents only included adults; no young people under 18 years old were included. Given that exposure to e-devices begins early in life and that young people rapidly adopt e-devices, the inclusion of young people in future studies would provide a better understanding of the associations between the pattern of e-device use and acute and chronic mental health problems. Finally, all data were self-reported, which might be subject to recall bias and social desirability bias. Future studies should consider including objective data on e-device use and adverse health effects.

### Conclusions

We provided the first findings of the associations of a shorter duration to the first e-device use after waking and a longer duration of e-device use before sleeping with high risks of anxiety and depressive symptoms in adults. Although further studies are required to examine causal relations, these two simple measures could help identify and manage e-device users with high risks for anxiety and depression.

## Abbreviations

ANOVA: analysis of variance
FHInTS: Family and Health Information Trends Survey
GAD-2: Generalized Anxiety Disorder-2
ICT: information and communication technology
PHQ-2: Patient Health Questionnaire-2
PHQ-4: Patient Health Questionnaire-4
PR: prevalence ratio
WHO: World Health Organization
